# Viscum pleurodesis is as effective as talc pleurodesis and tends to have less adverse effect

**DOI:** 10.1007/s00520-020-05405-0

**Published:** 2020-03-12

**Authors:** YongJin Chang, DeogGon Cho, KyuDo Cho, MinSeop Cho

**Affiliations:** Department of Thoracic and Cardiovascular Surgery, St. Vincent’s Hospital, College of Medicine, The Catholic University of Korea, Seoul, Republic of Korea

**Keywords:** Malignant pleural effusion, Chemical pleurodesis, Talc, Viscum

## Abstract

**Purpose:**

Many patients diagnosed with advanced cancer have malignant pleural effusion that does not respond to chemotherapy or radiation therapy. These patients often have respiratory symptoms, especially dyspnea. In order to relieve these symptoms, various procedures including chemical pleurodesis have been performed. Although talc is the most widely used and effective sclerosing agent, there it has various adverse effects. The objective of this study was to determine whether Viscum **(**ABNOVA Viscum**®** Fraxini Injection, manufactured by ABNOVA GmbH, Germany) could be used as an agent to replace talc in clinical practice.

**Methods:**

Data of 56 patients with malignant pleural effusion who received chemical pleurodesis after tube thoracostomy from January 2003 to December 2017 were retrospectively reviewed to analyze clinical course and response after pleurodesis with each agent.

**Results:**

After pleurodesis, changes in numeric rating scale (NRS) was 1.4 ± 1.6 in the talc group and 0.5 ± 1.5 in the Viscum group (*p* = 0.108). Changes in white blood cell counts after pleurodesis were 4154.8 ± 6710.7 in the talc group and 3487.3 ± 6067.7 in the Viscum group (*p* = 0.702). Changes in C-reactive protein (CRP) were 9.03 ± 6.86 in the talc group and 6.3 ± 7.5 in the Viscum group (*p* = 0.366). The success rate of pleurodesis was 93.3% in the talc group and 96% in the Viscum group (*p* = 0.225).

**Conclusion:**

Viscum pleurodesis showed comparable treatment results with talc pleurodesis while its adverse effects such as chest pain and fever tended to be relatively weak.

## Introduction

The ultimate goal of treatment for patients with malignant pleural effusion (MPE) is to increase quality of life during life expectancy. These patients often have respiratory symptoms. Repeat thoracentesis, pig tailed catheter drainage, or tube thoracostomy is often used to relieve patient’s symptoms. However, such procedure itself can degrade the quality of life. Therefore, chemical pleurodesis is generally recommended to help patients feel at ease. In the past and now, talc is generally used as a sclerosing agent for chemical pleurodesis [1, 2]. Many researchers have made efforts to select sclerosing agents that are as effective as talc. Unfortunately, previous studies have focused on the “final therapeutic outcome” of chemical pleurodesis using talc and several sclerosing agents without focusing on “patient’s discomfort” during pleurodesis.

The authors introduced Viscum **(**ABNOVA Viscum**®** Fraxini Injection, manufactured by ABNOVA GmbH, Germany) as an alternative agent for talc and applied it to patients. The purpose of this study to compare the final therapeutic outcome of chemical pleurodesis using talc and Viscum based on various values related to clinical course. The usefulness of Viscum in chemical pleurodesis for patients with MPE was also determined.

## Materials and methods

After obtaining institutional review board approval from the Catholic University of Korea, a retrospective review was conducted of 56 consecutive patients with MPE who received chemical pleurodesis after tube thoracostomy on bedside at St. Vincent’s Hospital, South Korea from January 2003 to December 2017. The medical records of each patient were reviewed and patients’ characteristics were collected. Before and after the procedure, drainage count, vital sign, lab data, pain score as NRS, and finding of plain chest x-ray and chest CT were investigated to analyze clinical course and response after chemical pleurodesis in each agent. The response rate in pleural effusion was assessed by plain chest x-ray examination within 4 weeks after the last pleurodesis [3].

### Talc slurry pleurodesis via chest tube

We injected 25 mg of pethidine intravenously prior to the procedure to patient for pain control. A dose of 2 g of sterile, asbestos-free talc (Steritalc® F2, manufactured by Novatech, France) mixed with 100 ml of sterile saline, was instilled through the chest tube, which was clamped for 2 h after the procedure. Patient’s position change was carried out for 2 h every 10 min. Plain chest x-ray was taken 30 min after the procedure was over. Chest drain was removed when chest radiograph confirmed satisfactory lung expansion and total 24-h drainage was less than 150 ml without air leak. Our policy was to observe at least 1 day after removing the chest tube. If there was no change in chest x-ray, the patient was discharged.

### Viscum pleurodesis via chest tube

A dose of 100 mg of Viscum mixed with 50 ml of sterile saline was instilled through the chest tube, which was clamped for 3 h after the procedure. The patient’s position change was carried out for 3 h every 10 min. Pain control and post-procedure progression were the same as those for talc slurry pleurodesis described above.

All procedures were performed in the same way. The procedure was performed under local anesthesia using 2% lidocaine on bedside. The chest tube was inserted with 28Fr in the case of “total white-out” and 24Fr for others.

### Statistical analysis

All descriptive data were expressed as frequency and mean ± standard deviation. Frequencies were compared using x^2^ or Fisher’s exact test for categorical variables and independent two-sample *t* test for continuous variables. The IBM SPSS software ver. 20.0 (IBM Corp., Armonk, NY, USA) was used for the analysis. Differences between groups were considered to be statistically significant when *p* value was less than 0.05.

## Results

### Patient characteristics

Among a total of 56 patients, thirty patients underwent talc pleurodesis and twenty-six patients underwent Viscum pleurodesis. Only those patients whose lung reexpansion was confirmed on chest x-ray after tube thoracostomy were included. Characteristics of each patient group are shown in Table 1. There were no significant differences in patient characteristics between the two groups except for male gender (talc vs. Viscum: 16 vs. 5, *p* = 0.009) and positive cytology (21.7% vs. 72.7%, *p* = 0.001).

**Table 1:** Patient characteristics before pleurodesis (*n* = 57)

	Talc (*n* = 30)	Viscum (*n* = 27)	*P* value
Age (year)	60.2 ± 17.9	64.9 ± 14.6	0.328
Gender (M)	16 (53.3%)	4 (23.5%)	0.047
Type of cancer			0.305
Lung cancer	8 (26.7%)	7 (25.9%)	
Breast cancer	8 (26.7%)	9 (33.3%)	
Ovarian cancer	2 (6.7%)	6 (22.2%)	
Others	12 (40%)	5 (18.5%)	
Total drainage (ml)	2883 ± 2104	3298 ± 1466	0.433
Body temperature (°C)	36.6 ± 0.3	36.5 ± 0.3	0.342
Pain (NRS)	1.4 ± 1.1	1.6 ± 1.0	0.503
WBC (cells/mm^3^)	7101.7 ± 2927.2	8482.4 ± 2978.2	0.134
CRP (mg/dL)	9.62 ± 8.85	5.16 ± 4.67	0.163
White-out on x-ray,%	5 (16.7%)	10 (58.8%)	0.334
Location of effusion (Rt.)	19 (63.3%)	8 (47.1%)	0.278
Follow up (m)	5.4 ± 8.1	2.8 ± 3.3	0.217

*NRS* numeric rating scale, *WBC* white blood cell, *CRP* C-reactive protein

### Clinical findings after talc pleurodesis

Body temperature (high fever within 2 days), WBC, and CRP were elevated after talc pleurodesis. Body temperature increased from 36.6 ± 0.3 to 37.7 ± 0.7 °C, WBC increased from 7101.7 ± 2927.2 to 12,046 ± 6208.3 cells/mm^3^, and CRP increased from 9.62 ± 8.85 to 16.09 ± 9.04 mg/dL. The degree of pain measured by NRS was increased from 1.4 ± 1.1 to 2.7 ± 1.3. After talc pleurodesis, it took 4.6 days to remove the chest tube. Total drainage amount was 476.0 ± 601.4 ml.

### Clinical findings after Viscum pleurodesis

Viscum pleurodesis also showed similar pattern to talc. Body temperature increased from 36.7 ± 0.4 to 37.7 ± 0.6 °C, WBC increased from 8450.8 ± 2679.5 to 14,109 ± 5203.3 cells/mm^3^, and CRP also increased from 4.75 ± 4.35 to 12.3 ± 6.6 mg/dL. The NRS rose from 2.0 ± 1.5 to 2.5 ± 1.7. It took 5.2 ± 4.2 days to remove the chest tube. Total drainage amount was 756.0 ± 1048.4 ml (Table 2).

**Table 2:** Study outcomes after pleurodesis

	Talc (*n* = 30)	Viscum (*n* = 27)	*P* value
Duration of drainage after tube thoracostomy (day)	11.5 ± 8.1	12.9 ± 6.1	0.495
Duration of drainage after pleurodesis (day)	4.6 ± 4.7	6.0 ± 5.0	0.345
Total drainage after pleurodesis (ml)	476.0 ± 601.4	1047.6 ± 1321.7	0.05
Body temperature (high fever within 2 days)	37.7 ± 0.7	37.6 ± 0.5	0.579
Pain(NRS)	2.7 ± 1.3	2 ± 1.3	0.154
WBC (cells/mm^3^)	12,046.3 ± 6208.3	13,820.8 ± 6414.2	0.416
CRP (mg/dL)	16.09 ± 9.04	9.90 ± 7.25	0.092

*NRS* numeric rating scale, *WBC* white blood cell, *CRP* C-reactive protein

### Changes of clinical features before and after pleurodesis between the two groups

Body temperature was increased 1.1 ± 0.7 °C in the talc group and 1.0 ± 0.6 °C (*p* = 0.699) in the Viscum group. WBC was increased 4154.8 ± 67,110.7 cells/mm^3^ in the talc group and 3487.3 ± 6067.7 cells/mm^3^ (*p* = 0.702) in the Viscum group. CRP was increased 9.03 ± 6.86 mg/dL in the talc group and 6.3 ± 7.5 mg/dL (*p* = 0.366) in the Viscum group. NRS was increased 1.4 ± 1.6 in the talc group and 0.5 ± 1.5 in the Viscum group (*p* = 0.108) (Table 3).

**Table 3:** Difference between before and after pleurodesis

	Talc	Viscum	*P* value
Body temperature (°C)	1.1 ± 0.7	1.1 ± 0.5	0.902
Pain (NRS)	1.4 ± 1.6	0.4 ± 1.3	0.092
WBC (cells/mm^3^)	4154.8 ± 6710.7	2086.5 ± 6285.2	0.3
CRP (mg/dL)	9.03 ± 6.86	4.39 ± 8.26	0.199

### Imaging results of treatment response in both groups within 4 weeks after the last pleurodesis

Regarding the final response after pleurodesis, 36.7% in the talc group and 60.0% in the Viscum group showed complete responses, 56.7% in the talc group and 36.0% in the Viscum group showed partial responses, and 6.7% in the talc group and 4.0% in the Viscum group showed treatment failure.

### Complications

There was one acute respiratory distress syndrome (ARDS) and one pneumonitis in the talc group and one ARDS in the Viscum group. There was no death due to pleurodesis.

## Discussion

The goal of performing chemical pleurodesis in patients with MPE is palliation, not definite treatment. Patients who suffer from dyspnea or chest pain are subject to discomfort due to repetitive hospitalization and treatment, resulting in reduced quality of life. Treatment of MPE includes repetitive thoracentesis, indwelling pleural catheter, and other methods [4–6]. However, chemical pleurodesis is still the most common treatment [7–11]. Generally, chemical pleurodesis can be performed through video-assisted thoracic surgery (VATS) or a chest tube at the bedside. However, VATS has potential risks associated with general anesthesia and surgery. Therefore, in the case of physician’s judgment or patient’s request, chemical pleurodesis is often performed at the bedside.

Agents used for chemical pleurodesis can vary, including tetracycline, minocycline, doxycycline, and iodopovidone. [2, 6, 7, 12, 13]. Traditionally, talc is known to be the most effective agent. The success rate of talc pleurodesis ranges from 81 to 100% [2, 7, 8, 14–16]. On the other hand, there have been various reports on the success rate of Viscum pleurodesis [17–19]. Cho et al. have reported that 96.7% had overall response rate, and 79% had complete response rate after chemical pleurodesis using ABNOVA Viscum**®** Injection for MPE [17]. In the results of this study, there was no statistically significant difference in the success rate of chemical pleurodesis between the talc group and the Viscum group (93.7% vs. 96.0%, *p* = 0.225).

Talc pleurodesis has been associated with minor complications such as pain and fever, and major complications such as lung injury [20–24]. Since respiratory failure and death have also been reported in pleurodesis using a small amount of talc [25], the authors used only 2 g of talc to prevent respiratory failure. Thus, there has been continuing effort to find agents that are as effective as talc while having fewer complications than talc. In this study, the authors compared various clinical outcomes during the course of chemical pleurodesis performed with talc and *Viscum album* extract for MPE. Viscum was first used in chemical pleurodesis in 1977 [26]. Basic mechanisms involved in chemical pleurodesis are quite different from between talc and Viscum. The most crucial difference is that talc induces inflammation while Viscum stimulates antitumor immunity. Viscum is also known to induce inflammation [27–33]. Because of this mechanism, this study also showed that the duration of drainage of the pleural fluid tended to be relatively long in the Viscum group after pleurodesis [17, 30]. However, the common discomfort experienced by most patients with chemical pleurodesis is pleuritic pain or fever. In relation to chemical pleurodesis, the authors want to learn more about “discomfort” such as fever and chest pain that patients most often experience.

In this study, we evaluated the degree of pain suffered by patients using the numeric rating scale (NRS) [34, 35]. Although this is a study of patients with pneumothorax, Song et al. have reported that only 13.5% of the Viscum group have complaints of severe pain (*p* = 0.006), and the mean value of NRS is 3.9 ± 2.2 after pleurodesis with Viscum [36]. However, we wanted to not only see the absolute value of NRS but also change patterns of values after chemical pleurodesis. According to our results, the talc group and the Viscum group showed similar results in NRS after the pleurodesis (2.7 ± 1.3 vs 2.5 ± 1.7, *p* = 0.7). However, the Viscum group showed smaller change in NRS between before and after the procedure (talc vs. Viscum, 1.4 ± 1.6 vs. 0.5 ± 1.5, *p* = 0.108). The degree of “discomfort” felt by patients with pleurodesis was not statistically different between the two groups, although patients in the Viscum group were relatively less afflicted.

Besides pain, the common discomfort felt by patients after pleurodesis is fever. According to previous studies of Froudarakis et al. based on the rise in WBC count and CRP levels, fever is caused by systemic inflammation after talc pleurodesis [20]. In this regard, the authors also analyzed changes in body temperature and inflammatory factors such as WBC and CRP. All three were statistically insignificant, although their changes in the Viscum group were smaller.

The intensity of the pain felt by patients and the degree of change in measured values related to the fever were similar between the two groups. However, patients in the Viscum group showed relatively less pain and less fever than the talc group, indicating a tendency to withstand relatively comfortable procedures.

This study has several limitations. First, the accuracy of medical record is questionable. In particular, due to the limitations of the retrospective study, there was no way to assess pain other than NRS, and this was often missing. In addition, some past medical records of patients were missing. From a technical point of view, the position of the chest tube might have affected the outcome, since pleurodesis was done at the bedside.

In conclusion, Viscum pleurodesis showed comparable treatment results to talc pleurodesis. However, its adverse effects such as chest pain and fever tended to be relatively weak.
